# Fiber Wavelength Meter Based on Fizeau Interferometer on wFBG for Phi-OTDR Signal Drift Compensation

**DOI:** 10.3390/s25247543

**Published:** 2025-12-11

**Authors:** Konstantin V. Stepanov, Tatyana V. Gritsenko, Roman I. Khan, Kirill I. Koshelev, Fedor L. Barkov, Andrey A. Zhirnov, Alexey B. Pnev

**Affiliations:** 1 Laser and Optoelectronic Systems Department, Radio Electronics and Laser Technology Faculty, Bauman Moscow State Technical University, 2-nd Baumanskaya 5-1, 105005 Moscow, Russia; chobantv@yandex.ru (T.V.G.); khan.roman.igorevich@gmail.com (R.I.K.); koshelev-k@yandex.ru (K.I.K.); a.zh@bmstu.ru (A.A.Z.); pniov@bmstu.ru (A.B.P.); 2 Institute of Continuous Media Mechanics of the Ural Branch of the Russian Academy of Sciences, Academician Korolev St. 1, 614013 Perm, Russia; barkov.f@permsc.ru

**Keywords:** optical fiber, wavelength meter, fiber interferometer, weak fiber Bragg gratings (wFBG), Mach-Zehnder interferometer (MZI), Michelson interferometer, Fizeau-based interferometer

## Abstract

The paper studies the characteristics of a wavelength meter (WLM) based on a Fizeau-based interferometer (FI) using weak Fiber Bragg Gratings (wFBGs). The proposed WLM is compared with the commercial Angstrom WLM, as well as with a Mach-Zehnder interferometer (MZI) based WLM. The error characteristics and applicability of the new WLM with different bases in wFBG pairs were analyzed.

## 1. Introduction

Nowadays, the accurate measurement and monitoring of laser wavelength drift is extremely important due to the rapid development of optical measurement technology. This is driven primarily by the strong demand for accuracy and stability in laser-based systems for various applications, including telecommunications [1], metrology [2,3], and scientific research [4].

Current technological advances allow for the production of commercially available C-band lasers with linewidths of less than 1 kHz [5,6,7,8]. Such highly stable light sources are essential components of fiber sensing systems, as they enable interferometric measurements of signal phase with an error of hundredths of a radian or even less [9,10,11,12]. However, such a narrow linewidth does not completely eliminate long-term frequency drifts in the laser source. Furthermore, this drift prevents the effective use of interferometer-based measurements for monitoring slow-changing disturbances, such as structural deformations (in bridges, towers, roads, and other structures) over a day or a season. To measure and compensate for such phase drifts, specialized wavelength meters (WLMs) can be used, such as those from HighFinesse [13] or Yokogawa [14]. However, these devices are often expensive, bulky, and in some cases, overly complex for the application. For phase drift monitoring, fiber interferometers such as the Mach-Zehnder interferometer (MZI) or Michelson interferometer (MI) are commonly used [15,16]. A key limitation of these interferometers, however, is their sensitivity to temperature (Δ*T*) and vibration (Δx), which cannot be fully eliminated even with large enclosures [17,18,19,20].

As expression (1) for the MZI’s phase difference shows, the effects of temperature or vibrations cannot be separated from the measured signal. Furthermore, it is impossible to determine the origin of a signal change or to distinguish between different influencing factors. Most significantly, the contribution of external disturbances to signal changes cannot be separated from the influence of the laser’s wavelength drift. It is worth noting that for other interferometers, the form of expression (1) remains similar, differing only by a coefficient accounting for the double-pass nature of the interferometer’s arms.

(1) $$∆\varphi \left(t\right)=\frac{2\pi \left(x+∆x\left(t\right)\right)\left(1+k\cdot ∆T\left(t\right)\right)}{\lambda \left(t\right)}$$

where ∆*φ*(*t*) is phase difference at the moment of *t*, *x* and ∆*x* is interferometer’s base and its changes in time, *k* is coefficient of thermal expansion for optical fiber, ∆*T* is the interferometer’s temperature changes, *λ* is laser linewidth in time.

This work proposes a Fizeau-based interferometer (FI) using weak fiber Bragg gratings (wFBGs) [21,22,23,24] to track laser wavelength drift over time. Compared to the MZI and MI, the proposed scheme has several advantages. First, it contains no bulk components (such as splitters or mirrors) other than the fiber itself, which makes it significantly less sensitive to external vibrations. Second, its all-fiber structure makes it more compact and significantly facilitates temperature stabilization and control to mitigate thermal effects. In our experiments, this was achieved using a thermostat that fits within a volume of 10 × 10 × 10 sm^3^ [25].

The aim of this work is to develop and study methods for measuring the wavelength drift of a laser source using fiber interferometers and to create a system that ensures high measurement accuracy and stability under various operating conditions [26,27,28,29,30,31]. This task is relevant and important, as highlighted by several research groups [32,33,34,35], but several questions remain unresolved.

## 2. Theory

To effectively measure the wavelength drift of a laser source, it is necessary to evaluate various fiber interferometer schemes.

This study requires a comparative analysis of different interferometer types capable of detecting phase shifts and, subsequently, measuring the wavelength of a laser source.

**Figure 1:**
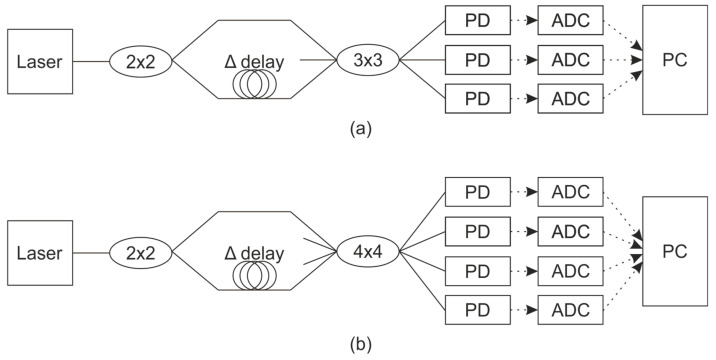
The MZI scheme for phase shift detection with: (**a**) 3 × 3 output coupler, (**b**) 4 × 4 output coupler. Here and in another schemes in manuscript: solid lines between elements are optic fiber and dashed lines arrows are electrical connection.

One of the most common interferometric schemes that can be used to detect phase shifts is the Mach-Zehnder interferometer (MZI). This scheme is shown in Figure 1a. 

The laser radiation is split into two beams by the first fiber-optic splitter. For optimal interference contrast, the split ratio between the two arms should be 50/50 (i.e., identical). The two beams then recombine and interfere at the second fiber-optic splitter. It is worth noting that the phase relationship between the outputs depends on the specific design and number of ports in the splitter:

For 2 × 2 output coupler:

(2) $$\left[\begin{array}{c} I_{PD1}\left(t\right)=I_{1}+I_{2}+2\sqrt[{}]{I_{1}\cdot I_{2}}\cdot \cos\left(2\frac{\pi }{\lambda }\Delta L\left(t\right)+\varphi_{0}\right)\;\\ I_{PD2}\left(t\right)=I_{1}+I_{2}+2\sqrt[{}]{I_{1}\cdot I_{2}}\cdot \cos\left(2\frac{\pi }{\lambda }\Delta L\left(t\right)+\varphi_{0}+\pi \right) \end{array}\right.$$

For 3 × 3 output coupler,

(3) $$\left[\begin{array}{c} I_{PD1}\left(t\right)=I_{1}+I_{2}+2\sqrt[{}]{I_{1}\cdot I_{2}}\cdot \cos\left(2\frac{\pi }{\lambda }\Delta L\left(t\right)+\varphi_{0}\right)\;\\ I_{PD2}\left(t\right)=I_{1}+I_{2}+2\sqrt[{}]{I_{1}\cdot I_{2}}\cdot \cos\left(2\frac{\pi }{\lambda }\Delta L\left(t\right)+\varphi_{0}+2\pi /3\right)\\ I_{PD3}\left(t\right)=I_{1}+I_{2}+2\sqrt[{}]{I_{1}\cdot I_{2}}\cdot \cos\left(2\frac{\pi }{\lambda }\Delta L\left(t\right)+\varphi_{0}-2\pi /3\right) \end{array}\right.$$

For 4 × 4 output coupler [36],

(4) $$\left[\begin{array}{c} I_{PD1}\left(t\right)=I_{1}+I_{2}+2\sqrt[{}]{I_{1}\cdot I_{2}}\cdot \cos\left(2\frac{\pi }{\lambda }\Delta L\left(t\right)+\varphi_{0}\right)\;\\ I_{PD2}\left(t\right)=I_{1}+I_{2}+2\sqrt[{}]{I_{1}\cdot I_{2}}\cdot \cos\left(2\frac{\pi }{\lambda }\Delta L\left(t\right)+\varphi_{0}+\pi /2\right)\\ I_{PD3}\left(t\right)=I_{1}+I_{2}+2\sqrt[{}]{I_{1}\cdot I_{2}}\cdot \cos\left(2\frac{\pi }{\lambda }\Delta L\left(t\right)+\varphi_{0}+\pi \right)\\ I_{PD4}\left(t\right)=I_{1}+I_{2}+2\sqrt[{}]{I_{1}\cdot I_{2}}\cdot \cos\left(2\frac{\pi }{\lambda }\Delta L\left(t\right)+\varphi_{0}+3\pi /2\right) \end{array}\right.$$

Consequently, the formulas for phase calculation, the possibility of phase unwrapping, and the associated errors differ among these designs. For 2 × 2 and 4 × 4 interferometers, a fundamental limitation is the inability to reliably determine the direction of a phase change when the phase reaches the limits of the [−π, +π] range, leading to ambiguity. In contrast, a 3 × 3 interferometer provides a solution to this problem. The formula for calculating the phase shift in a 3 × 3 interferometer is given by [37].

(5) $$\Delta \varphi \left(t\right)=arctan\left(\frac{\sqrt[{}]{3}\left(I_{PD1}\left(t\right)-I_{PD3}\left(t\right)\right)}{2I_{PD2}\left(t\right)-I_{PD1}\left(t\right)-I_{PD3}\left(t\right)}\right)$$

A significant disadvantage of this MZI scheme for laser wavelength measurement is its high sensitivity to external factors, particularly temperature fluctuations and vibration. This sensitivity persists even when using bulky vibration-isolating enclosures.

**Figure 2:**
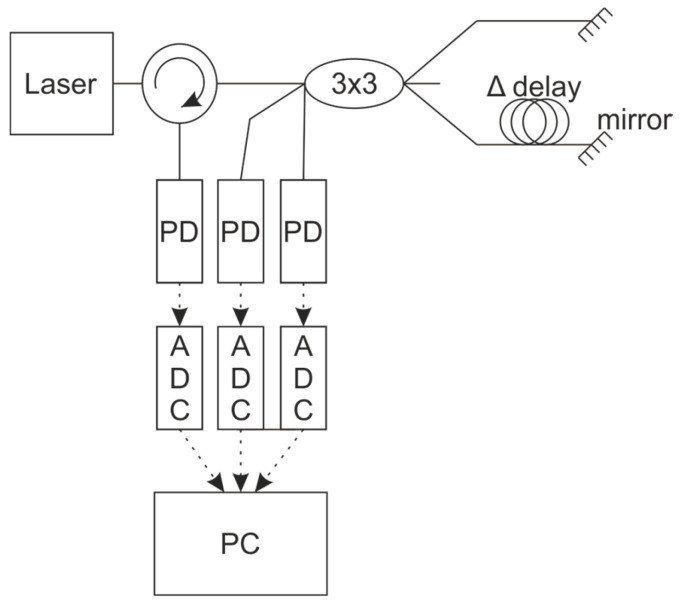
The MI scheme for phase shift detection.

An alternative approach is based on the Michelson interferometer (MI) scheme, which is a classic two-beam interferometer. In its bulk-optics form, a light beam is split by a beam splitter, while in its fiber-optic implementation, this is achieved using a splitter and circulators, as shown in Figure 2. Conceptually, the MI can be characterized as a folded MZI, where the signal is reflected back by a pair of mirrors.

This double-pass configuration compensates for polarization changes within the interferometer, even without dedicated polarization-preserving fiber, and effectively halves the optical path difference between the arms. The phase shift equations for 2 × 2, 3 × 3, and 4 × 4 interferometers, along with their respective phase unwrapping formulas, are also applicable here. However, a drawback is that we must either use one fewer detector or incorporate a circulator. Using a circulator necessitates additional matching of the fiber lengths from the splitter to each receiver. Furthermore, the MI shares the same fundamental disadvantages described earlier for the MZI.

The Fizeau interferometer (FI) differs fundamentally from the MI and MZI in that it lacks distinct arms. It operates using a single light beam reflected from two partially reflective mirrors arranged in sequence. In our implementation, these “mirrors” are wFBGs. Although a circulator is required to divert the reflected radiation for detection, the interferometer’s base and sensitive element are integrated into a single segment of fiber without any splitters. This design makes the interferometer significantly more stable against vibrations. The all-fiber construction also enables a more compact form factor. The output signal of the FI can be represented as follows:

(6) $$I_{PD}\left(t\right)=I_{1}+I_{2}+2\sqrt[{}]{I_{1}\cdot I_{2}}\cdot cos\left(4\frac{\pi }{\lambda \left(t\right)}\Delta L\left(t\right)+\varphi_{0}\right)$$

It can be seen that the coefficient in the argument of the cosine function for the phase is 4 instead of 2. This results from the double passage of radiation through the section of fiber between the wFBGs.

**Figure 3:**
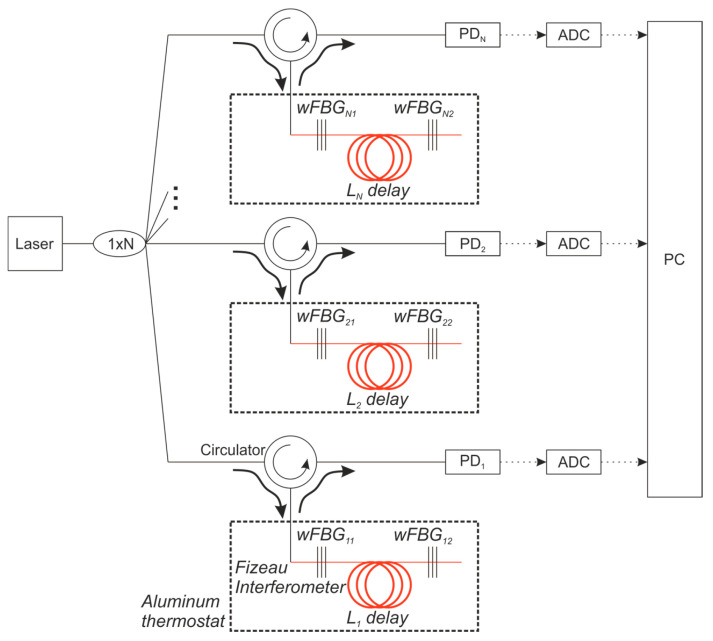
The FI scheme for phase shift detection.

However, the lack of a splitter at the FI output makes direct phase unwrapping impossible. Therefore, alternative methods are required to track the phase across period transitions. One such method is to use several FIs with different bases connected in parallel. An example of this scheme is shown in Figure 3.

For this FI scheme configuration, unwrapping can be performed as follows:

(7) $$\left[\begin{array}{c} I_{PD1}\left(t\right)=I_{1}+I_{2}+2\sqrt[{}]{I_{1}\cdot I_{2}}\cdot \cos\left(4\frac{\pi }{\lambda \left(t\right)}\Delta L_{1}\left(t\right)+\varphi_{01}\right)\;\\ I_{PD2}\left(t\right)=I_{1}+I_{2}+2\sqrt[{}]{I_{1}\cdot I_{2}}\cdot \cos\left(4\frac{\pi }{\lambda \left(t\right)}\Delta L_{2}\left(t\right)+\varphi_{02}\right)\\ \ldots \\ I_{PDN}\left(t\right)=I_{1}+I_{2}+2\sqrt[{}]{I_{1}\cdot I_{2}}\cdot \cos\left(4\frac{\pi }{\lambda \left(t\right)}\Delta L_{N}\left(t\right)+\varphi_{0N}\right) \end{array}\right.$$

These formulas assume the following relationship between the interferometer bases: $\Delta L_{N}\ll ..\ll \Delta L_{2}\ll \Delta L_{1}$. In this case, the same change in the laser source wavelength, $∆\lambda \left(t\right)=\;\lambda \left(t+1\right)-\;\lambda \left(t\right)$ will have a markedly different effect on the phase in each interferometer, resulting in $\Delta \varphi_{N}\ll ..\ll \Delta \varphi_{2}\ll \Delta \varphi_{1}$. Consequently, the change in the photodiode signal will also follow the same hierarchy: $\Delta I_{PDN}\ll ..\ll \Delta I_{PD2}\ll \Delta I_{PD1}$. To eliminate the ambiguity of the unwrap procedure, the following steps are necessary:

1. Determine Specifications. Define the required operating range $\Delta \lambda_{tot}$ and the maximum permissible error $\Delta \lambda_{err}$ for the laser wavelength fluctuation meter.
2. Select the Longest Base (*L*_1_). Based on the error value ($\Delta \lambda_{err}$), select the largest interferometer base length, ${\Delta L}_{1}$. This is performed using the following evaluation expression, which guarantees that the minimum detectable phase change is resolved to at least 1/10 of the full scale by the ADC:
  $$\begin{array}{l} 4\frac{\pi }{\lambda }\Delta L_{1}-4\frac{\pi }{\lambda +\Delta \lambda_{err}}\Delta L_{1}>0.1\pi \\ \Delta L_{1}>\frac{0.1\lambda^{2}}{4\Delta \lambda_{err}} \end{array}$$
3. Add Shorter Bases. According to the required operating range ($\Delta \lambda_{tot}$), add interferometers with successively shorter base lengths, each roughly an order of magnitude smaller than the last. Continue this until the shortest baseline, $\Delta L_{N}$, satisfies the condition that laser tuning across the entire operating range causes a phase change of less than π/4, preventing initial ambiguity:
  $$\begin{array}{l} 4\frac{\pi }{\lambda }\Delta L_{N}-4\frac{\pi }{\lambda +\Delta \lambda_{tot}}\Delta L_{N}<\pi /4\\ \Delta L_{N}<\frac{\lambda^{2}}{16\Delta \lambda_{tot}} \end{array}$$
4. Coarse measurement. To reconstruct the phase signal, it is necessary to begin with the interferometer with the shortest base ($\Delta L_{N}$). Its phase will not contain ambiguities within the full operating range, providing a coarse but unambiguous measurement.
5. Refining measurements. Subsequently, reconstruct the phase sequentially from each interferometer with a longer base. Use the unambiguous phase trace from the previous (shorter) interferometer to resolve the 2π ambiguities in the current one. This process gradually increases the resolution and accuracy of the measurement along the laser’s frequency axis.

Thermal stabilization and vibration protection.

The authors hypothesized that to minimize the effects of temperature and vibration, it is necessary to reduce both the spatial dimensions and the mass/length of the fiber within the interferometer. Reducing the spatial dimensions can be achieved by downsizing the components, for example, by implementing the interferometer on a waveguide plate [38,39,40,41,42]. A significant problem with this approach is the high cost, as manufacturing a plate with the required path difference of several meters or tens of meters would be prohibitively expensive. An alternative, proposed in this paper, is to use a Fizeau interferometer (FI) with weak Fiber Bragg Gratings (wFBGs). This design does not require splitters or Faraday mirrors. Consequently, the mass (and inertia) of the interferometer is reduced to that of a few meters of fiber, ensuring an order of magnitude lower sensitivity to external mechanical vibrations and enabling a more compact design. In this study, the fiber was mounted in a trench of an aluminum thermostat with a radius of 4 cm. This setup ensures high temperature stability for the entire interferometer base and allows for convenient monitoring and adjustment. The aluminum thermostat is equipped with a temperature sensor attached to the trench walls and a heating element for active control. Special attention was paid to the reflectivity, R, of the wFBGs. The chosen value must satisfy two main requirements.

Firstly, they must reflect sufficient radiation to be detected by a photoreceiver with a bandwidth of ~MHz (NEP on the level of 0.1–10 pW/√Hz) and a saturation power of <100 µW.

Secondly, they must reflect a small enough portion of light to eliminate second and higher-order multipass interference, the strongest of which (2nd order) is proportional to *R*^2^. To achieve a Signal-to-Noise Ratio (SNR) of 30 dB or higher under these conditions, a reflectivity of R ≈ 1% is required. In our experiments, we used R = 0.3%. An additional advantage of wFBGs compared to standard FBGs is their broad reflection spectrum (on the order of a few nm). This reduces the strong dependence of reflected intensity on wavelength drift and enables operation over a larger wavelength range.

Temperature stabilization can be achieved using active or passive approaches, each with distinct advantages and disadvantages. Active temperature control maintains a setpoint outside the ambient range, which simplifies the feedback loop operation. However, this forces the loop to continuously generate a correction signal. While the loop’s frequency response can be adjusted to eliminate noticeable oscillations, the system remains sensitive to minute temperature fluctuations. Given the optical fiber’s linear expansion coefficient of ~7 × 10^−6^, even a temperature variation of 0.005 K will induce a phase shift in π, representing significant noise. Furthermore, the error of the temperature sensor and the adjustment accuracy of the feedback loop typically exceed this value.

Under such conditions, passive temperature control appears more attractive. While it does not limit the overall range of temperature change as active control does, it does not introduce noise from corrective thermal shifts, which is a more valuable characteristic for this measurement scheme.

Mathematical simulation.

To theoretically analyze the performance of the proposed scheme, a computer model was created with the following main parameters, and is shown in Table 1.

This model is based on Formula (1) and was used to investigate the system’s error dependencies on various combinations of the parameters listed above. The following key conditions were considered for the simulation series:

1. The setup is intended for sensor systems utilizing lasers with a narrow linewidth (<1 MHz) but potentially significant frequency drift, ranging from a few MHz per minute to hundreds of MHz per millisecond. Measuring the laser linewidth itself requires interferometers with long delays [6], which is beyond the scope of this article. In our scheme, a maximum arm length difference on the order of tens of meters allows for tracking laser frequency drift, where the total measurable range is determined by the shortest base, and the measurement error is determined by the longest.
2. In the proposed wFBG-based interferometer scheme, vibrations are minimized because the path difference exists only within a low-inertia fiber spool. Therefore, the contribution of vibration is considered negligible, a point that will be confirmed by experimental studies in the following section.
3. Under passive thermal stabilization, temperature fluctuations are expected to be on the order of tenths of a degree over tens of seconds due to the thermal inertia of the housing. A critical aspect is the resolution of the temperature sensor, for which modern sensors can achieve thousandths of a degree. Smoothing time filters can also be employed, as temperature is a slowly varying parameter.
4. The signal SNR is determined by the photoreceiver quality and ADC quantization, which directly influences system cost. This study analyzed several component combinations, and the results for these will be presented.

**Figure 4:**
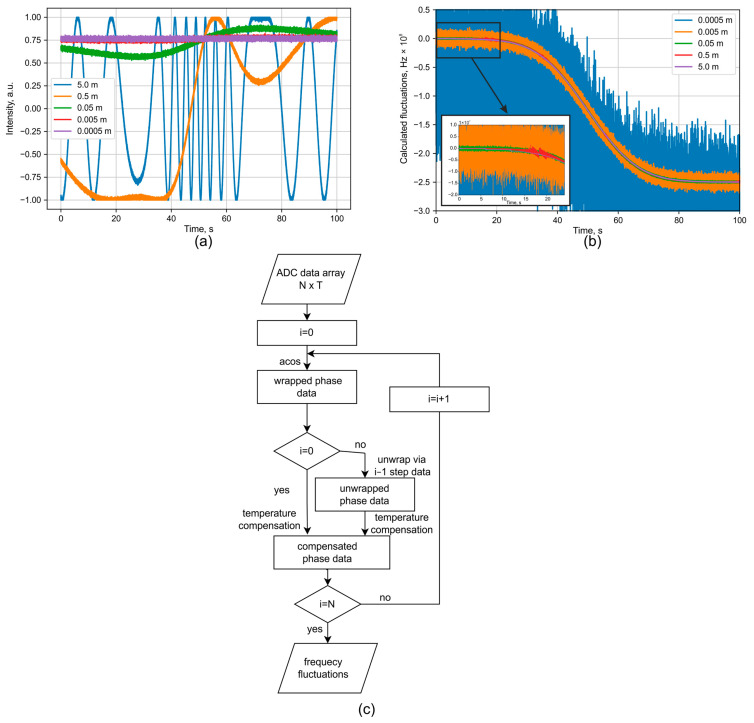
Plots of (**a**) the recorded ADC signal, (**b**) the decoded frequency fluctuations with zoomed initial region on inset, and (**c**) decoding algorithm.

An example of signal simulation is shown in Figure 4.

A signal was simulated to include a temperature drift of 0.2 degrees during the measurement, with added noise corresponding to a specified SNR of 20 dB. Phase unwrapping was performed sequentially from the shortest to the longest baseline. The results demonstrate that for baselines of 0.05 m or less, the phase does not cross the upper or lower range boundaries, thereby avoiding ambiguity during the unwrapping of phases from longer baselines. These longer baselines are crucial for reducing the measurement error. This is evident in Figure 4b, where the interferometer with a 5 m baseline achieves an error of less than 3 MHz. The algorithm used to recalculate the interferograms into frequency drift is presented in Figure 4c. The initial measurement data consist of T interferograms, with N points in each. The time axis t can be generated at any stage based on the ADC sampling frequency, *fs*, using the relation *t* = *N*/*fs*. The processing cycle begins with the interferogram from the shortest interferometer base. For this data, the number of oscillations is relatively small, and the phase can be calculated directly using a simple acos(*data*) function. Next, the calculated phase is compensated for the temperature-induced phase shift. This compensation is calculated using Equation (1), under the condition that the vibrational component is zero (∆*x* = 0). This cycle is then repeated for each subsequent interferogram (*i*-th) from the next shortest base to the longest. A key addition in this loop is a phase unwrapping step. As the distance between wFBGs increases, the phase oscillations become more frequent and will eventually reach the uncertainty limits of ±π. At these points, the direction of the phase change (increasing or decreasing) is determined by referencing the phase direction from the previously unwrapped (*i* − 1)-th interferogram at the same point in time. Finally, the total unwrapped phase change is recalculated into the laser frequency drift. It can be described with the following Equation (8):

(8) $$∆\nu \left(t\right)=\frac{∆\varphi \left(t\right)}{4\pi nx/c}-\frac{k\cdot ∆T\left(t\right)}{\lambda \left(t\right)n/c}$$

From this formula it is possible to directly calculate frequency error if its initiating error is presented in radians of measured phase or degrees of measured temperature. For interferometer base 5 m and standard parameters of SMF presented in Table 1, we can highlight some values for more clear understanding: Δ*ν*(*t*) ≈ ·(∆*φ*(*t*)/π)·10^7^ − ∆*T*(*t*)·10^8^.

Temperature and frequency errors.

**Figure 5:**
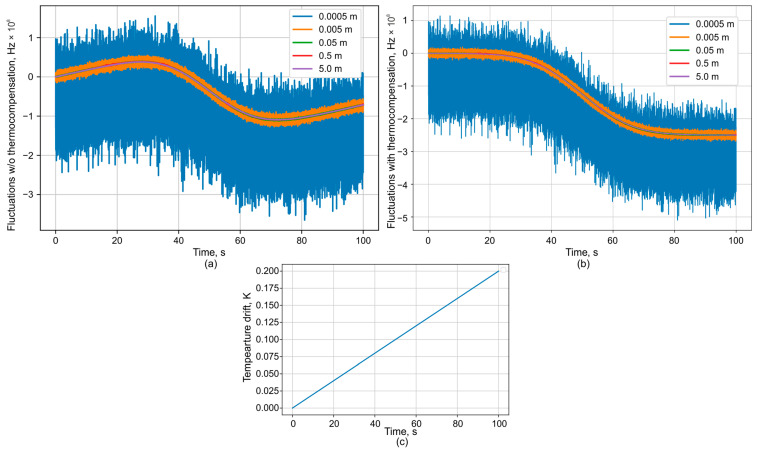
Plots of the decoded frequency fluctuations (**a**) without and (**b**) with (**c**) measured temperature drift.

It is important to note that the influence of temperature on the interferometer readings is inseparable from the actual temperature fluctuations of the device. Therefore, electronic temperature sensors are used to measure these fluctuations directly within the thermostat. The error of these sensors is less than 0.01 K. Given the smooth nature of thermal drift, this allows for effective interpolation and smoothing of the temperature record. Consequently, the temperature-induced phase shift can be compensated to a level where its residual error is lower than the error contributions from photodetector noise and phase unwrapping. Previous studies conducted for Raman OTDR (distributed temperature sensor system), as shown by the authors in [25], demonstrated that a fiber segment shorter than 100 m can be thermally stabilized with a peak-to-peak accuracy of approximately 0.04 K. Moreover, it was confirmed that the temperature drift is very slow. This slow drift allows for the error to be further reduced through time averaging, thereby increasing the overall accuracy. The example of wavelength drift measurements with and without temperature fluctuation compensation is given in Figure 5.

**Figure 6:**
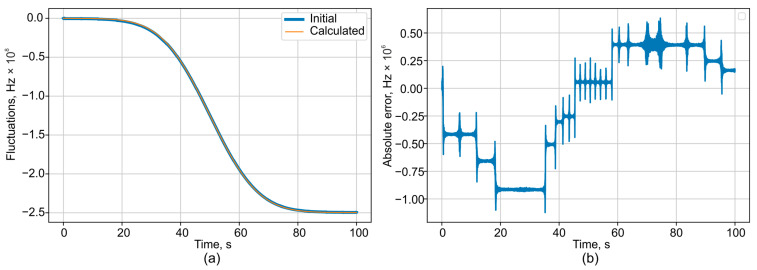
(**a**) Comparison plots of the decoded and initial frequency fluctuations and (**b**) absolute error of decoded result (difference between simulated input and calculated).

In this case, the random error obtained from processing and phase unwrapping for the interferometer with the longest base is within ±1.5 MHz, excluding any systematic components. The corresponding data are presented in Figure 6. For error quantification in subsequent computer simulations, the maximum absolute value from the error plot, an example of which is shown in Figure 6, will be used.

**Figure 7:**
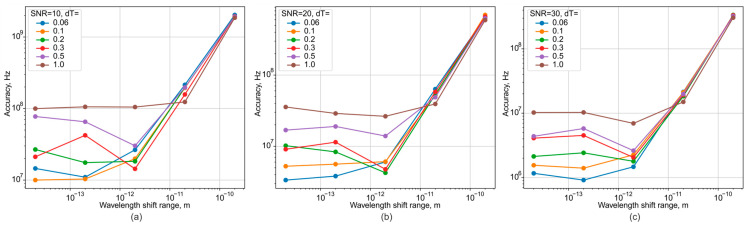
Dependence plots of the wavelength measurement error on the SNR ((**a**) 10 dB, (**b**) 20 dB, (**c**) 30 dB), tuning range and temperature.

A series of simulations was conducted to determine the wavelength measurement error as a function of system parameters, specifically signal-to-noise ratio (SNR) and the level of temperature fluctuations. The results are presented in Figure 7.

This simulation modeled the wavelength of the radiation source tuning from one value to another, as is typical during the operation of a source within measurement or other optical systems. The main conclusions drawn from the resulting graphs are as follows.

The SNR requirements can be set at a minimum of 20 dB. This enables wavelength fluctuation measurements with an error of less than 10 MHz, which is an acceptable level comparable to most commercially available devices. Consequently, the proposed module can achieve similar performance in a more compact form factor and at a significantly lower cost. An SNR of 20 dB corresponds to one significant noise sample per 100 ADC samples or, for a 14-bit ADC, approximately 16 noise counts. This is a feasible target and provides a margin to compensate for noise in the optical components. It should also be noted that with an optimal optical assembly and a higher SNR, the error can be reduced to a few MHz, which is sufficient for tracking the drift of narrow-linewidth lasers with a linewidth of up to 100 kHz.

**Figure 8:**
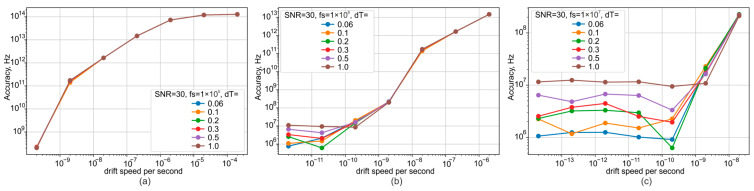
Dependence plots of the wavelength measurement error on ADC sampling rate (**a**) 100 kHz, (**b**) 1 MHz, (**c**) 10 MHz) and frequency drift rate.

With a wide laser source tuning range, the measurement error can accumulate in a stepwise fashion, as shown in Figure 6. This error depends on the instantaneous frequency tuning rate of the laser. It can be mitigated by increasing the ADC sampling rate to the megahertz range. However, since this scheme is intended as a frequency drift tracking module for systems with fixed laser source parameters, the sampling rate can be selected based on a priori knowledge of the source’s maximum tuning rate. Graphs of the fluctuation measurement error under these conditions are shown in Figure 8.

The graphs demonstrate that increasing the sampling frequency allows for a proportional reduction in measurement error. This is because the change in source frequency per sample is a critical variable for system performance. The 10 MHz error limit was chosen as a target to enable the use of a relatively inexpensive ADC. The dependence of measurement accuracy on temperature stability is also significant. As shown in Figure 8c, for high values of frequency drift rate relative to the ADC sampling frequency, temperature variations are not the limiting factor. However, their impact becomes critical when the drift is small and the potential error is on the order of 10 MHz or lower. For a 1 MHz sampling rate, this effect becomes noticeable for frequency drifts lower than 100 pm/s. As demonstrated in [25], the thermostabilization module used can maintain temperature with a peak-to-peak error of approximately 0.04 K. Therefore, we can expect the frequency error caused by temperature fluctuations to be on the order of a few MHz.

## 3. Experiment

**Figure 9:**
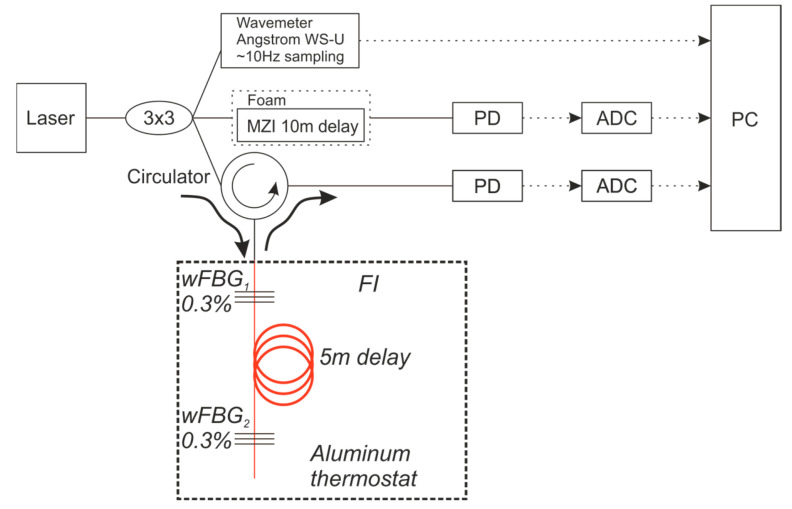
The experimental scheme in which the signals from the MZI and the FI were compared with each other, as well as with the reference WLM.

Experiments were conducted to compare the signals from the MZI and the proposed FI with each other and with a reference Angstrom WS-U wavelength meter. The experimental setup is shown in Figure 9. The output from an NKT Basik X15 laser source was split into three paths using a 3 × 3 splitter. The first path was connected to the reference WLM. The second path was directed to an MZI, which was housed inside a foam-lined box and had a 10 m arm length difference. The third path was routed through a circulator to the FI. The FI’s fiber, which contained the wFBGs (with a reflectivity of 0.3% and a separation of 5 m), was entirely housed within an aluminum thermostat. No additional damping materials were used for the FI. The wavelength readings from the reference WLM, which has a specified accuracy of 2 MHz, were sent directly to a PC. The signals from both interferometers were detected by 400 kHz photodetectors (PD), digitized by an ADC with a sampling frequency of 100 kHz, and then sent to the PC for final processing.

This experiment allowed for a comparison of the temperature stability and vibration resistance between the MZI and the FI with equivalent arm length differences under identical conditions. The effects of the external environment on the reference WLM and the interferometers were observed simultaneously. Since interferometers can form the basis of an all-fiber wavelength meter, this setup enabled a direct comparison of their stability. It is important to note that this comparison involved a single interferometer as the WLM sensing element, not a set of interferometers, and was based on the magnitude of phase drift over the same time period.

**Figure 10:**
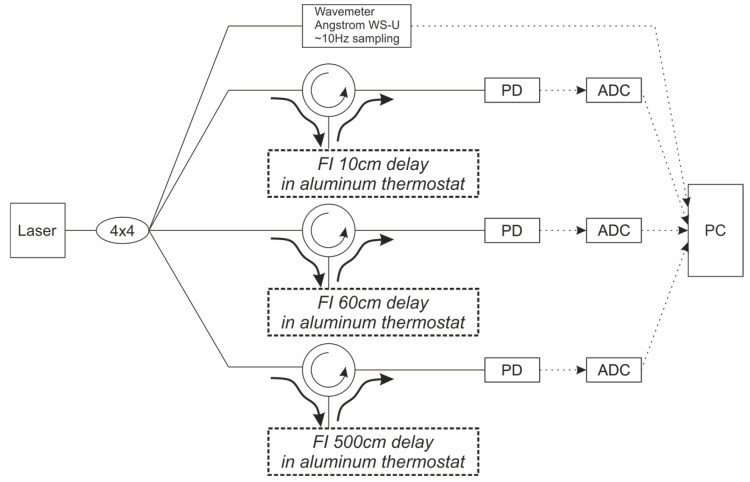
Experimental scheme with different bases of FI and with the reference WLM.

The objective of the second experiment was to confirm the feasibility of not only recording phase drift but also directly measuring the wavelength using several FIs with different spacings between the wFBGs. The experimental setup is shown in Figure 10. The output from the NKT Basik X15 laser source was split into four paths using a 4 × 4 splitter. The first path was connected to the reference WLM. Each of the remaining three paths was routed through a circulator to a different FI, with wFBG spacings of 10 cm, 60 cm, and 500 cm. All wFBGs had a reflectivity of 0.3%. The output from each interferometer was sent to a dedicated PD, digitized by an ADC, and finally processed on a PC alongside the data from the reference WLM.

This setup functions as a prototype for an all-fiber WLM. Each branch with a different interferometer base acts as an independent WLM sensor. When the wavelength tunes, it introduces a distinct phase shift in each sensor, which can be used to calculate the absolute wavelength shift.

In addition to confirming the feasibility of wavelength measurement using this array of FIs, the design allows us to evaluate how environmental conditions similarly affect FIs with different base lengths and to establish the measurement error limits for an all-fiber WLM.

## 4. Analysis and Discussion

Based on the experimental results, we would like to highlight two key points demonstrating the advantages of the proposed FI.

**Figure 11:**
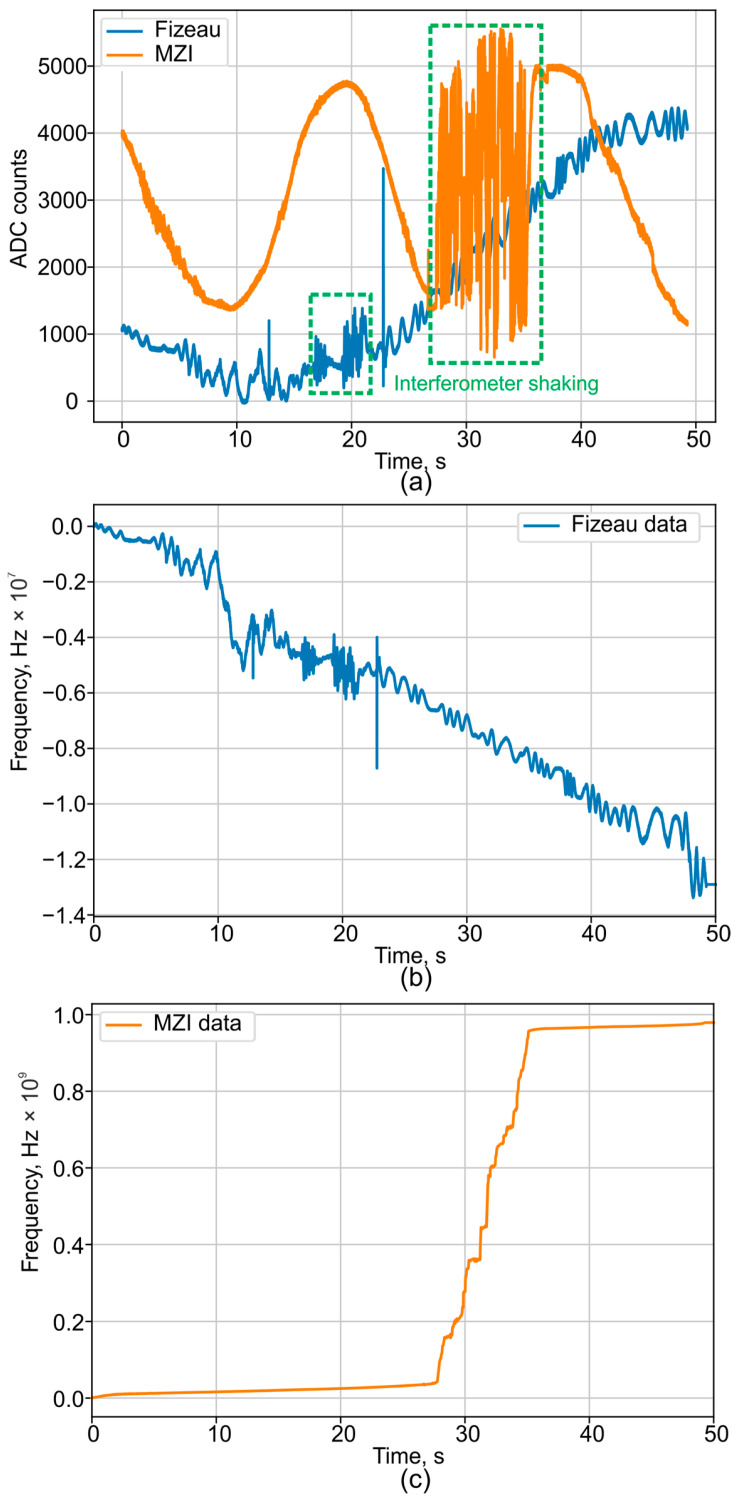
The advantages demonstration of the FI vibration insensitivity for external shaking events: (**a**) raw interferograms, (**b**) phase drift of FI, and (**c**) phase drift of MZI.

First, the MZI proved to be significantly more sensitive to external mechanical vibrations. Figure 11 shows that under identical external vibrations, the phase of the FI deviates by approximately 1 rad (without the interferogram signal crossing the ±π boundaries), whereas the phase of the MZI deviates by tens of radians, resulting in multiple phase wrapping transitions. This comparison was made for an equivalent optical path difference of 10 m in both interferometers (a 10 m arm length difference for the MZI and a 5 m base with double-pass for the FI). The higher sensitivity of the MZI is attributed to the influence of the external environment on its two spatially separate fiber arms, making it impossible to fully isolate them from acoustic and vibrational noise. In contrast, the FI’s signal is more stable because the interfering beams travel through a common fiber path, allowing for the compensation of phase shifts induced by external vibrations.

**Figure 12:**
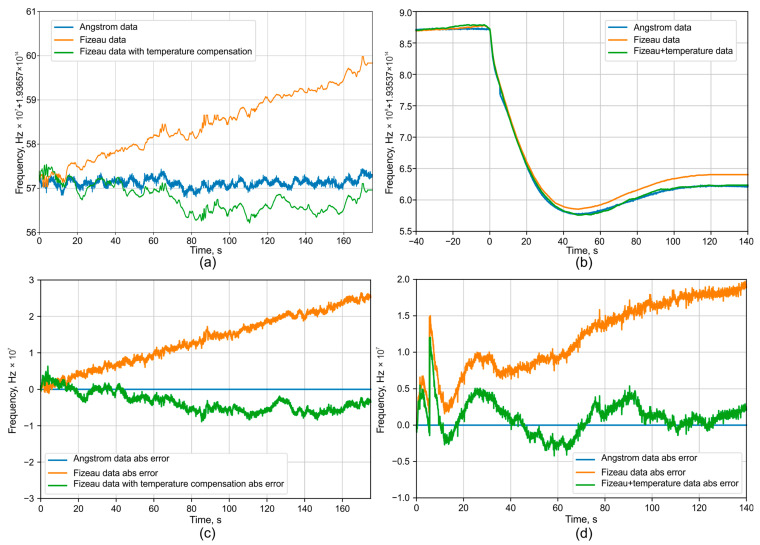
Temperature compensation demonstration of the FI and comparison with an absolute WLM: (**a**) stable regime, (**b**) 2 pm wavelength shift using software, (**c**) error for stable regime, and (**d**) error for shifting.

Second, it is demonstrated that by measuring the interferometer’s temperature, its effect on frequency fluctuation measurements can be compensated for according to Equation (1). As a result, the measurement error of the FI relative to the reference WLM is significantly reduced, as presented in Figure 12. The residual error is largely determined by the accuracy and resolution of the temperature measurement system. The experimental error obtained corresponds to the simulation results shown in Figure 8, and its absolute value does not exceed the expected limit.

Figure 11a provides a qualitative comparison of the MZI’s and FI’s sensitivity to vibrations. For a more detailed analysis, the signals from the FI (Figure 11b) and the MZI (Figure 11c) can be compared. It is evident that the frequency oscillations in the FI are on the order of a few MHz. In contrast, the MZI exhibits an apparent frequency drift on the order of GHz, which is severe enough to disrupt the entire measurement procedure.

**Figure 13:**
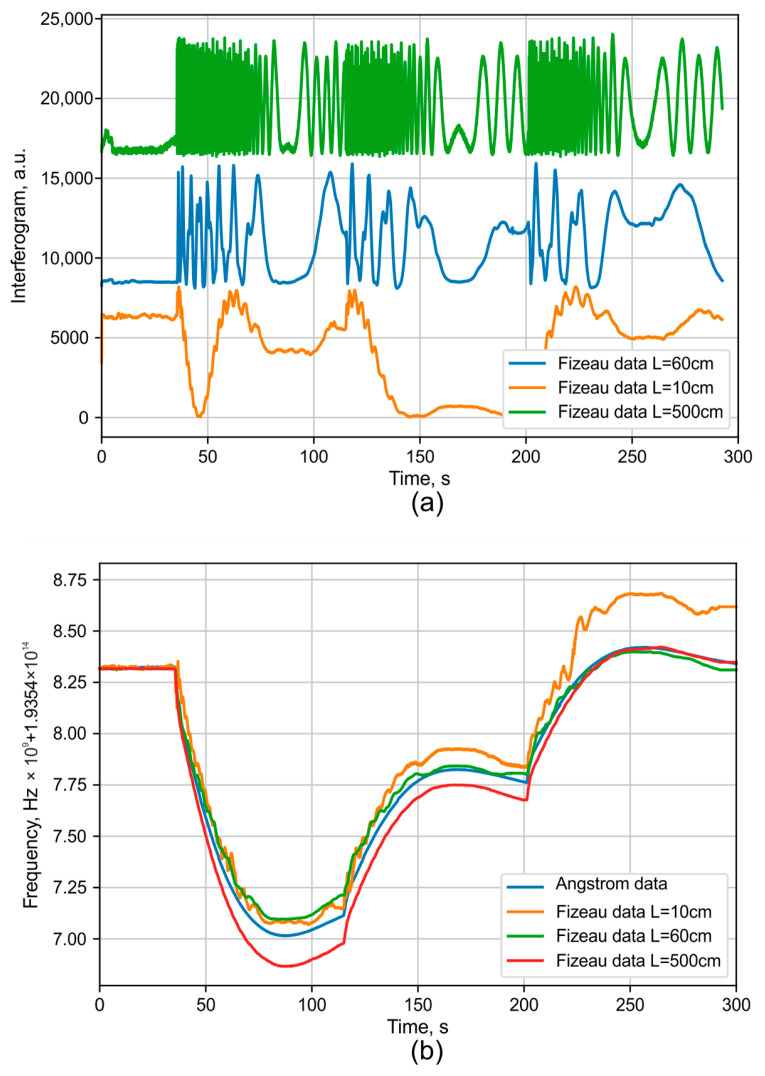
Wavelength selection demonstration using interferometers with different bases: (**a**) raw signal, (**b**) processed data without thermal compensation.

The coarse and fine measurements of wavelength drift using interferometers with different delay lengths are presented in Figure 13. It is evident that the interferometer with the shortest delay (10 cm) has significantly lower sensitivity to wavelength drift. The data from this short-delay interferometer can be used to determine the primary direction of the drift, while the data from the 60 cm and 500 cm delay interferometers are used to refine these results with progressively higher resolution.

The experiments also revealed that polarization drift of the laser radiation has a significant effect. The Jones matrix formalism [43] provides a method for describing how the polarization of a light wave changes as it propagates through an optical fiber. In this formalism, a coordinate system is selected. The Jones vector XY at a given point in space describes the polarization state of the wave at that point, namely

$$E=X\cdot E_{1}+Y\cdot E_{2},$$

where E is the electric field strength of the wave, E_1_ and E_2_ are unit base vectors. The fiber is divided into separate homogeneous elements [44], each of which has its own transformation matrix M_i_, and the change in polarization of the light wave as it passes through an element is determined by the relationship

X′Y′=MiXY

where XY is the Jones vector at the input to the element, X′Y′—one at the output. Thus, the change in a wave’s polarization state as it propagates through a section of optical fiber is described by a Jones matrix that is the product of the matrices representing the individual elements within that section.

It is important to note that, in an ideal case without any defects, a single-mode fiber is a homogeneous medium, and its Jones matrix is unitary, meaning it preserves the polarization state of the input radiation. However, the presence of random imperfections alters this behavior. The primary source of polarization defects in single-mode fiber (SMF) is birefringence caused by cylindrical inhomogeneities. In such a defective element, the refractive indices n_1_ and n_2_ for the two orthogonal polarizations of the light wave are not equal. In a coordinate system aligned with the principal axes of this birefringent element, its Jones matrix M has the form:

$$M=\left(\begin{array}{cc} 1 & 0\\ 0 & exp(-i∆\phi ) \end{array}\right),$$

where ∆φ=2πn1−n2Lλ is the phase delay, L is the length of the defective element, and λ is the wavelength. In the original coordinate system, the Jones matrix of an element has the form T(ψ) M T(−ψ), where T(ψ) =$\;\left(\begin{array}{cc} \cos\psi & \sin\psi \\ -\sin\psi & \cos\psi \end{array}\right)$ is the rotation matrix by angle ψ between the coordinate systems.

Since the values of *n*_1_ − *n*_2_, *L*, and *ψ* are random (though their distribution is determined by the fiber’s quality), the polarization state of radiation propagating through a standard single-mode fiber becomes randomized. The characteristic length for complete polarization misalignment ranges from several meters to several tens of meters [45]. From the perspective of the interference pattern, a change in the wave’s polarization state introduces an additional factor in the interference term of the equation (6). This factor is associated with the misalignment of the electric field vectors when the two waves are superimposed—a phenomenon known in our adopted terminology as polarization-induced fading:

$$\cos\theta =\left(X_{1}X_{2}+Y_{1}Y_{2}\right)/\sqrt{X_{1}X_{1}+Y_{1}Y_{1}}/\sqrt{X_{2}X_{2}+Y_{2}Y_{2}},$$

where *θ* is the angle between the strength vectors, X1Y1 and X2Y2 are the Jones vectors.

**Figure 14:**
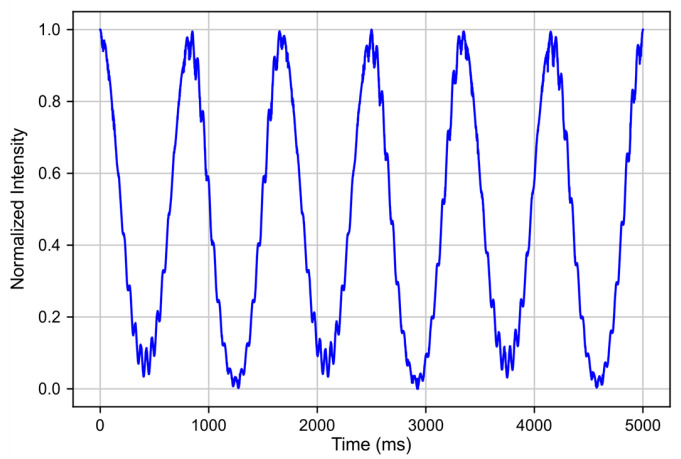
Example of simulated polarization beat effect on reflectogram which gives similar behavior to experimental data.

The simulated effect of polarization defects on the interference pattern is shown in Figure 14. In the experimental data, this effect manifests as oscillations on the order of fractions of a radian in the interferogram.

In future work, we propose to suppress this noise by using polarization-maintaining (PM) fiber and minimizing the length of the fiber lead connecting the laser source to the wavelength meter’s interferometric sensor.

## 5. Conclusions

We have presented a C-band laser wavelength meter based on a Fizeau interferometer formed by weak Fiber Bragg Gratings. The proposed design demonstrates advantages in thermal and vibration stability. By compensating for thermally induced signal drift, the system achieves a measurement error of less than 3 MHz, as confirmed by experimental studies. This wavelength meter can serve as a reference to improve the accuracy of phase-sensitive Optical Time-Domain Reflectometers (phi-OTDRs) by precisely measuring laser wavelength drift. While similar goals have been pursued using other methods, such as twice-differential techniques [46] or DTGS systems [47], those approaches did not fully eliminate temperature effects. Therefore, the proposed module enables significant performance improvements for Distributed Acoustic Sensing (DAS) systems.

## Figures and Tables

**Table 1:** Parameters of computer model.

Symbol	Value	Description
Constants		
N	100,000	number of time samples in the simulation
fs	1000	sampling frequency
k	6.92 × 10^−6^	coefficient of thermal expansion of SMF28
lam_0	1550.12 × 10^−9^	meters, initial wavelength
c	3 × 10^8^	speed of light in vacuum
n_eff	1.5	refractive index of fiber
Variables		
snr_db	20	SNR in dB
dx	0	external influence on the fiber, variable
dT_max	0.2	degC—total shift in temperature, variable
lam_max	3 × 10^−12^	meters, wavelength shift, variable
x_arr	np.array ([5, 0.5, 0.05, 0.005, 0.0005])	array of distance between wFBGs—bases of interferometers

## Data Availability

The data presented in this study are available on request from the corresponding author.

## Abbreviations

The following abbreviations are used in this manuscript:

FI	Fizeau-based interferometer
MI	Michelson interferometer
MZI	Mach-Zehnder interferometer
wFBG	weak fiber Bragg grating
WLMs	wavelength meter
